# Effect of implementing a heart failure admission care bundle on hospital readmission and mortality rates: interrupted time series study

**DOI:** 10.1136/bmjqs-2022-015511

**Published:** 2023-11-05

**Authors:** Thomas Woodcock, Dionne Matthew, Raffaele Palladino, Mable Nakubulwa, Trish Winn, Hugh Bethell, Stephen Hiles, Susan Moggan, Jackie Dowell, Paul Sullivan, Derek Bell, Martin R Cowie

**Affiliations:** 1 School of Public Health, Faculty of Medicine, Imperial College London, London, UK; 2 Strategy, Guy’s and St Thomas’ Hospital NHS Foundation Trust, London, UK; 3 Life Science, LOGEX BV, Amsterdam, Netherlands; 4 Department of Public Health, University of Naples Federico II School of Medicine and Surgery, Naples, Italy; 5 Research and Development, London Northwest University Healthcare NHS Trust, London, UK; 6 Cardiology, London Northwest University Healthcare NHS Trust, London, UK; 7 Business Development, PHASTAR Specialist Biometric Contract Research Organisation, London, UK; 8 Royal Brompton Hospital & School of Cardiovascular Medicine, Faculty of Lifesciences & Medicine, King’s College London, London, UK

**Keywords:** Healthcare quality improvement, Evaluation methodology, Implementation science

## Abstract

This study aimed to evaluate the impact of developing and implementing a care bundle intervention to improve care for patients with acute heart failure admitted to a large London hospital. The intervention comprised three elements, targeted within 24 hours of admission: N-terminal pro-B-type natriuretic peptide (NT-proBNP) test, transthoracic Doppler two-dimensional echocardiography and specialist review by cardiology team. The SHIFT-Evidence approach to quality improvement was used. During implementation, July 2015–July 2017, 1169 patients received the intervention. An interrupted time series design was used to evaluate impact on patient outcomes, including 15 618 admissions for 8951 patients. Mixed-effects multiple Poisson and log-linear regression models were fitted for count and continuous outcomes, respectively. Effect sizes are slope change ratios pre-intervention and post-intervention. The intervention was associated with reductions in emergency readmissions between 7 and 90 days (0.98, 95% CI 0.97 to 1.00), although not readmissions between 0 and 7 days post-discharge. Improvements were seen in in-hospital mortality (0.96, 95% CI 0.95 to 0.98), and there was no change in trend for hospital length of stay. Care process changes were also evaluated. Compliance with NT-proBNP testing was already high in 2014/2015 (162 of 163, 99.4%) and decreased slightly, with increased numbers audited, to 2016/2017 (1082 of 1101, 98.2%). Over this period, rates of echocardiography (84.7–98.9%) and specialist input (51.6–90.4%) improved. Care quality and outcomes can be improved for patients with acute heart failure using a care bundle approach. A systematic approach to quality improvement, and robust evaluation design, can be beneficial in supporting successful improvement and learning.

**WHAT IS ALREADY KNOWN ON THIS TOPIC**

- Internationally, clinical guidelines clearly specify best practice care for patients admitted to hospital with acute heart failure. However, attempts to reliably translate these guidelines into practice have varying degrees of success and are often poorly evaluated.

**WHAT THIS STUDY ADDS**

- This study demonstrates how a robust approach to implementation, coupled with a strong evaluation design, can support successful improvement in quality of acute heart failure care, and ensure learning is captured.

**HOW THIS STUDY MIGHT AFFECT RESEARCH, PRACTICE OR POLICY**

- Acute hospital providers wishing to improve quality of acute heart failure care should consider adopting the heart failure admission care bundle approach. Teams evaluating quality improvement initiatives should consider using an interrupted time series with controls design where appropriate.

## Introduction

Heart failure (HF) affects approximately 64 million people around the world as of 2017, with health expenditure around 108 billion US dollars.^1 2^ This figure is likely to continue to increase with an ageing population. In the UK, around 900 000 people have HF, accounting for up to 2% of total National Health Service (NHS) expenditure.^3^ HF symptoms, including breathlessness, ankle swelling and fatigue, often result in decreased quality of life leading to debility, morbidity and mortality.^4^ The cost of ideal HF treatment focusing on optimal drug therapy appears to be reasonable uses of healthcare resource.^5^ The implementation of both device and drug therapies has improved the effectiveness of HF care along with the continuous development of advanced outpatient disease management strategies.^6^ Despite this, the prognosis for patients with HF remains poor with the multinational European Society of Cardiology and Heart Failure Association HF long-term registry figures showing in-hospital mortality at 5.5% and 1-year all-cause mortality at 26.7%.^7^

The UK National HF Audit 2013–2014 made clear recommendations for improvement in in-hospital HF care. These included focusing on implementation of the 2014 National Institute for Health and Care Excellence (NICE) clinical guidelines for HF, data quality, clinical coding of HF, specialist input to care and therapeutic treatment on discharge.^8^ As a result of these recommendations, in August 2014, a care pathway redesign initiative was launched at London North West University Healthcare NHS Trust (LNWUH), using a care bundle approach to provide key information to clinicians on recommended practice for patients with HF and collect data on care provided. A care bundle is a set of three to five evidence-based interventions designed to improve patient care when performed collectively.^9^ Success of a care bundle is often influenced by the support around the implementation process that enables its use in practice.^10^ The National Institute for Health Research Collaboration for Leadership in Applied Health Research and Care for Northwest London Programme provided support to this initiative through funding and quality improvement expertise.

The setting for this study was the LNWUH NHS Trust, one of the largest integrated healthcare trusts in England. It serves a diverse population of over 1 million people and provides hospital and community services to several London boroughs including Harrow, Brent and Ealing. The trust includes three hospital sites: Ealing Hospital (EH), Central Middlesex Hospital and Northwick Park Hospital (NPH).

## Methods

### Study aim and design

The aim of this study was to evaluate the LNWUH HF care bundle project against its key outcome measures. The improvement aim of this project was: ‘to improve the health, quality of life and experience of care for patients with acute HF in Northwest London at high value, and allow them to participate in their care in a way that suits them’.

An interrupted time series (ITS) design with control was used, with baseline period 1 January 2012–30 June 2015 and intervention period 1 July 2015, the date from which the care bundle was implemented into routine practice, to 31 July 2017. The control site was EH and the intervention site was NPH, both part of LNWUH NHS Trust. During the period of this study, the care bundle intervention was implemented at NPH but not implemented at EH. There were no other improvement initiatives focused on heart failure at EH during the study period.

### Outcomes

The primary outcome was rate of emergency readmission to hospital for any reason following discharge from an emergency admission with diagnosis of HF. This outcome was measured as rates of emergency readmission 7 days, 7–30 days and 30–90 days. Secondary outcomes for this same population were in-hospital mortality and length of stay in hospital. The latter was included as a balancing measure to reflect the concern that improving quality of care might delay discharge from hospital.^11^

### Data sources

Administrative data from the LNWUH data warehouse were extracted for all patient spells in hospital with discharge date within the period 1 January 2012–31 July 2017 inclusive (the study period). Data for spells at EH were only available from 1 April 2014. These data contained International Statistical Classification of Diseases and Related Health Problems 10th Revision (ICD-10) diagnosis codes documented post-discharge by LNWUH clinical coding department.^12^ Data on care bundle compliance were collected proactively by cardiac nurses identifying patients as in hospital with new-onset HF, or with acute decompensation of chronic HF. This was done at the ward handover morning meeting Monday–Friday during the implementation period using a paper form. These data were then entered into an Excel spreadsheet with data validation by an information analyst on a weekly basis. The analyst checked and remedied data quality issues on entry, using the electronic patient record.

### Study population

The population of interest was patients admitted to hospital as an emergency with a diagnosis of HF. The unit of observation for the study was a spell in hospital: in other words, a patient being admitted to hospital as an inpatient, staying in hospital for a period of time (the length of stay) and then being discharged home or to another location, or dying in hospital. Data were extracted on all emergency spells for the study period, and the study population defined as any such spell in which at least one diagnosis code recorded for the patient was one of the following: I110, I255, I420, I429, I500, I501, I509.

### Care bundle design and implementation

The care bundle was developed and piloted by a multidisciplinary team at NPH, using standard criteria for care bundle development.^9^ The team comprised consultant cardiologists, specialist cardiac nurses, a patient representative, quality improvement experts, data analysts and researchers. The bundle was developed over three stages. First, the team reviewed the evidence-based NICE guidance for acute HF,^13^ and the National HF Audit findings for NPH compared with national results.^8^ Second, the team proposed, discussed and agreed a set of elements for inclusion in the bundle, at a consensus meeting. The team agreed that the focus of this intervention would be on the admission process, rather than taking a care pathway approach encompassing the whole of the patient stay from admission to discharge. This was in part influenced by the available resources for the project, and in part by the necessity of improving diagnosis and identification of patients with HF before improvements in downstream care, such as appropriate medications, could be targeted. Finally, a paper form, to prompt clinicians to deliver the bundle and collect data on compliance, was iteratively tested in practice using the Plan–Do–Study–Act method.^14^

The team used the Collaboration for Leadership in Applied Health Research and Care Northwest London systematic approach, a quality improvement approach based on the SHIFT-Evidence framework, to support design, implementation and sustainability of the intervention.^15^ The quality improvement methods used are described in online supplemental appendix A. Particular attention was paid to the long-term success of the initiative, and a separate study conducted to explore risks to sustainability.^16^ Throughout the initiative, the team used the Web Improvement Support for Healthcare platform to collate, store and analyse data.^17^

10.1136/bmjqs-2022-015511.supp1 Supplementary data

Bundle implementation involved changing several processes and procedures to facilitate improved compliance with the elements of the bundle, for example, HF specialist nurses attending handover meetings on the acute medical unit to improve case ascertainment, initiate care bundles, get feedback, provide additional education as needed and promote best practice. The team also engaged and trained staff, including nurses and junior doctors, through induction sessions, and provision of feedback on their use of the bundle.

### Statistical analysis

Demographic characteristics for the study population were summarised and compared for the two sites and for the baseline and intervention periods using difference-in-difference analysis, through logistic and multinomial regression. To evaluate the impact of the intervention on the outcomes, we conducted ITS analysis with and without control for slope change, using mixed-effects Poisson regression for count outcomes and mixed-effects log-linear regression for continuous length of stay, the latter having a skewed distribution.^18 19^ Given that large instantaneous shifts in the outcomes were implausible, in the main analysis, we constrained the effect of time to be continuous across the interruption, using linear splines. We relaxed this constraint in a sensitivity analysis. In the analysis without control, the intervention effect was defined to be the (multiplicative) difference in trend pre-intervention and post-intervention on the intervention site. In the analysis with control, it was defined to be the (multiplicative) difference in difference in trend pre-intervention and post-intervention, on the intervention site compared with control. We report 95% CIs and corresponding p values for these effects. As some patients were admitted more than once to the same site over the study period, individuals were included as random effect in the regression model, while other covariates were included as fixed effect. A preliminary ITS analysis was conducted on all inpatient admissions at the intervention site, with count of spells with a diagnosis of HF as dependent variable, to check for any changes in coding before and after the intervention. To account for any potential differences in characteristics of patients admitted between the intervention and control sites and over the duration of the study, the following covariates were included in the regression models: gender, ethnicity (white/non-white), age (log-transformed to improve model fit), type of ward (acute assessment, cardiology, medicine, surgery, other) and month of the year to control for seasonality. Index of Multiple Deprivation (IMD) data were available as a measure of socioeconomic deprivation, but were missing not at random and hence were excluded from the main analysis. A sensitivity analysis was performed rerunning all models on a restricted dataset with IMD included as a covariate.

Analysis was conducted using R V.3.4.4 for data cleaning, linkage, descriptive statistics and ITS plots, and STATA V.15.0 for the ITS analysis.

## Results

### Care bundle intervention

The elements identified were as follows:

1. Diagnostic review of brain natriuretic peptide. N-terminal pro-B-type natriuretic peptide (NT-proBNP) measurement is recommended in NICE guidelines CG108 for patients with suspected HF.
2. Diagnostic referral for echocardiography. The guidelines also state that patients with new suspected acute HF should have transthoracic Doppler two-dimensional echocardiography performed within 24 hours of admission.
3. Specialist review by HF team. NICE guideline (CG187) recommends that patients admitted with suspected acute HF have early and continuing input from a dedicated specialist HF team. This is defined as being seen by a consultant cardiologist, another consultant with specialist HF interest or an HF specialist nurse.^4^

Echocardiography is the most economic and readily available method of imaging the heart to gather key information about both structure and function.^20^ Performing an echocardiogram within 48 hours of hospital admission for adults with new suspected acute HF enables earlier diagnosis and appropriate management of location of treatment, specialist input and pharmacological treatment.^21^ Previous research has demonstrated that patients with HF under specialist care received more evidence-based interventions and had better outcomes when compared with non-specialist care.^22–25^

### Quality improvement implementation

Mapping of the patient admission process revealed that most patients admitted with acute HF were admitted to the acute assessment wards. This led the team to initially focus implementation on these wards, rolling out to other ward areas as the project developed. This targeted approach enabled the cardiology specialist team to identify patients within the first 24 hours of admission and develop a process by which patients received the care bundle earlier in their admission.

The mapping of echocardiography processes identified that patients were waiting between 2 and 9 days to have inpatient echocardiography diagnostic tests completed. As a result, the team decided to set a standard that 90% of patients eligible for the care bundle should receive an echocardiogram within 24 hours. The process mapping also identified a gap in capacity and demand both in terms of workforce and equipment. A business case was developed to recruit two full-time stenographers and purchase four new echocardiograph machines, enabling rapid access to echocardiography.

**Figure 1:**
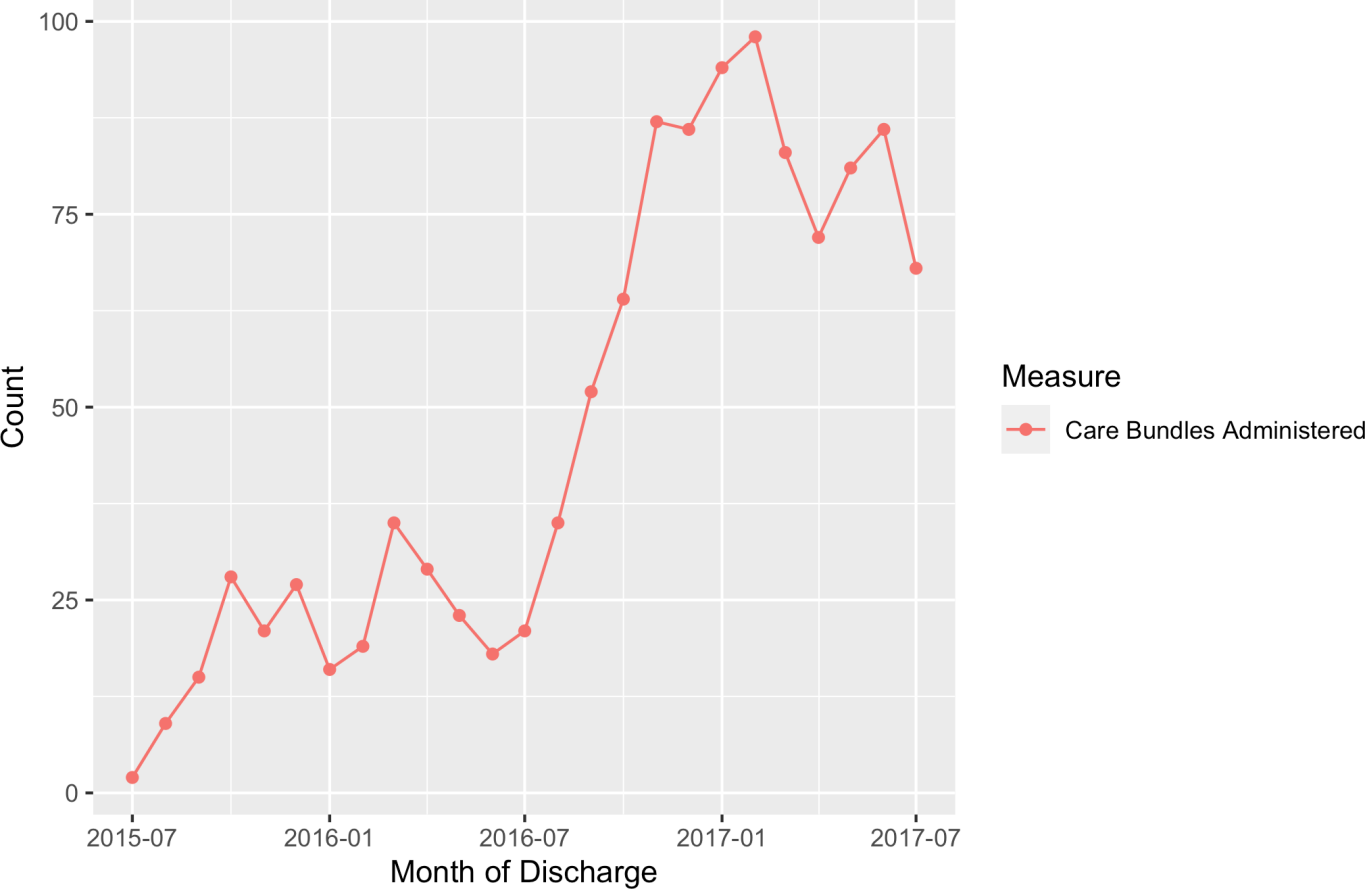
Number of care bundles administered per month, intervention site, June 2015–July 2017.

Implementation of the care bundle proceeded through weekly team meetings and review of bundle coverage data showing how many care bundles were implemented. The increase in bundle coverage over time is shown in figure 1. In total, between July 2015 and July 2017, 1169 care bundles were administered to patients with a recorded diagnosis of HF.

National audit data for the care bundle elements on the control and intervention sites are shown in table 1. These data show increasing numbers of patients submitted to the national audit by the intervention site, with a slight decrease in the high proportion receiving an NT-proBNP test. There were improvements in compliance with echocardiography and specialist input on the intervention site in the 2015–2017 period when compared with 2014/2015. While the proportion of patients with input from a consultant cardiologist decreased, the proportion with input from the specialist team increased by a larger amount.

**Table 1:** Compliance with care bundle elements

Site	Control site			Intervention site		
Year	2014/2015	2015/2016	2016/2017	2014/2015	2015/2016	2016/2017
No of admissions audited	283	240	402	163	705	1101
NT-proBNP-confirmed diagnoses*	280 (98.9%)	230 (95.8%)	402 (100%)	162 (99.4%)	673 (95.5%)	1082 (98.2%)
**With echocardiogram**†	270 (96.4%)	211 (91.7%)	356 (90.8%)	137 (84.6%)	666 (99.0%)	1070 (98.9%)
Admitted as cardiology inpatient†	141 (50.4%)	89 (38.7%)	104 (25.9%)	74 (45.7%)	261 (38.8%)	403 (37.2%)
With input from consultant cardiologist†	178 (63.6%)	96 (41.7%)	170 (42.3%)	75 (46.3%)	275 (40.9%)	397 (36.7%)
**With input from specialist team**†	213 (76.1%)	159 (69.1%)	271 (67.4%)	84 (51.9%)	562 (83.5%)	978 (90.4%)

National Heart Failure Audit results relevant to care bundle elements, control and intervention sites for financial years (commencing 1 April) 2014/2015–2016/2017. Bundle elements are highlighted in bold. Admission as a cardiology inpatient means the patient was admitted to a bed in a cardiology ward. Input from specialist team includes being seen by a consultant cardiologist, another consultant with specialist heart failure interest or a heart failure specialist nurse.

*NT-proBNP-confirmed diagnosis percentages are given out of number of admissions audited.

†Subsequent care item compliance percentages are given out of NT-proBNP-confirmed diagnoses.

NT-proBNP, N-terminal pro-B-type natriuretic peptide.

### Characteristics of the study population

A total of 15 618 spells for 8951 patients met the inclusion criteria, of which 1262 and 2553 spells were at the control site pre-intervention and post-intervention, and 6050 and 5753 spells were at the intervention site pre-intervention and post-intervention, respectively. Characteristics and outcomes of these spells are described in detail in table 2. The median age band of patients at the start of spell was 80–85 on both sites during both periods, and the age band distribution was skewed left. To within 1%, half of the spells were for women and half for men on both sites during both periods. A slightly greater proportion of spells on the intervention site, post-intervention, was for patients with Asian or Asian British or Mixed or other ethnic groups than pre-intervention. When compared with the smaller differences pre-intervention and post-intervention on the control site, the ORs for the difference-in-difference were 1.29 (95% CI 1.09 to 1.53) and 1.47 (95% CI 1.10 to 1.97), respectively, for these ethnicity categories. On the intervention site, the mean IMD score increased from 18.4 pre-intervention to 19.3 post-intervention, while remaining essentially constant on the control site. The resulting difference-in-difference of 1.2 (95% CI 1.05 to 1.36) was significant.

**Table 2:** Patient characteristics and outcomes for hospital spells included in the study

	Control site, post-intervention (N=2553)	Control site, pre-intervention (N=1262)	Intervention site, post-intervention (N=5753)	Intervention site, pre-intervention (N=6050)	Difference-in-difference OR/mean (95% CI)
Age					
Missing	0 (0.0%)	0 (0.0%)	0 (0.0%)	0 (0.0%)	
Median (IQR): band midpoints	82.5 (72.5–87.5)	82.5 (72.5–87.5)	82.5 (72.5–87.5)	82.5 (72.5–87.5)	0.91 (0.80 to 1.04)
Gender					
Missing	0 (0.0%)	0 (0.0%)	0 (0.0%)	0 (0.0%)	
Female	1293 (50.7%)	642 (50.9%)	2816 (49.0%)	3084 (51.0%)	1.07 (0.92 to 1.25)
Male	1260 (49.4%)	620 (49.1%)	2937 (51.1%)	2966 (49.0%)	
Ethnicity					
Missing	39 (1.5%)	10 (0.8%)	9 (0.2%)	17 (0.3%)	
Black or black British	193/2514 (7.7%)	106/1252 (8.5%)	526/5744 (9.2%)	480/6033 (8.0%)	1.30 (0.98 to 1.73)
Mixed/other ethnic groups/not stated	227/2514 (9.0%)	112/1252 (9.0%)	524/5744 (9.1%)	486/6033 (8.1%)	1.47 (1.10 to 1.97)
White	1136/2514 (45.2%)	544/1252 (43.5%)	2565/5744 (44.7%)	3007/6033 (49.8%)	
Asian or Asian British	958/2514 (38.1%)	490/1252 (39.1%)	2129/5744 (37.1%)	2060/6033 (34.2%)	1.29 (1.09 to 1.53)
Index of Multiple Deprivation					
Missing	138 (5.4%)	49 (3.9%)	775 (13.5%)	508 (8.4%)	
n; median (IQR)	2415; 25.6 (17.1–33.2)	1213; 25.85 (17.2–32.4)	4978; 17.7 (12.3–24.9)	5542; 16.7 (11.7–23.7)	
n; mean (SD)	2415; 25.6 (10.7)	1213; 25.8 (10.1)	4978; 19.3 (10.1)	5542; 18.4 (9.3)	1.20 (1.05 to 1.36)
Any-cause readmission category					
No readmission <90 days	1588 (62.2%)	774 (61.3%)	3908 (67.9%)	3976 (65.7%)	
<7 days	211/965 (21.9%)	96/488 (19.7%)	359/1845 (19.5%)	410/2074 (19.8%)	
≥7–<30 days	409/965 (42.4%)	198/488 (40.6%)	698/1845 (37.8%)	811/2074 (39.1%)	
≥30–<90 days	345/965 (35.8%)	194/488 (40.0%)	788/1845 (42.7%)	853/2074 (41.1%)	
Mortality					
Missing	0 (0.0%)	0 (0.0%)	0 (0.0%)	0 (0.0%)	
In-hospital mortality	243 (9.5%)	139 (11.0%)	550 (9.6%)	893 (14.8%)	
Length of stay					
Missing	0 (0.0%)	0 (0.0%)	0 (0.0%)	0 (0.0%)	
Median (IQR) days	7.5 (3.2–15.2)	8.8 (4.0–17.6)	6.7 (2.8–12.9)	7.1 (3.0–14.4)	

Demographic characteristics and unadjusted outcomes for patients discharged following an admission with heart failure. ORs are for difference-in-difference in each characteristic between sites across pre-intervention and post-intervention periods, calculated using logistic regression for gender, ordered logistic regression for age band and multinomial regression for ethnicity.

Readmission rates (crude, any cause, 7 days and 7–30 days) increased pre-intervention to post-intervention on the control site, and decreased on the intervention site. Readmissions between 30 and 90 days showed the reverse pattern. Crude mortality decreased on both sites, with a larger decrease on the intervention site (14.8% down to 9.6%) compared with control (11.0% down to 9.5%). Median length of stay also decreased on both sites, down from 8.8 to 7.5 days on the control site and from 7.1 to 6.7 days on the intervention site.

### ITS analyses

**Figure 2:**
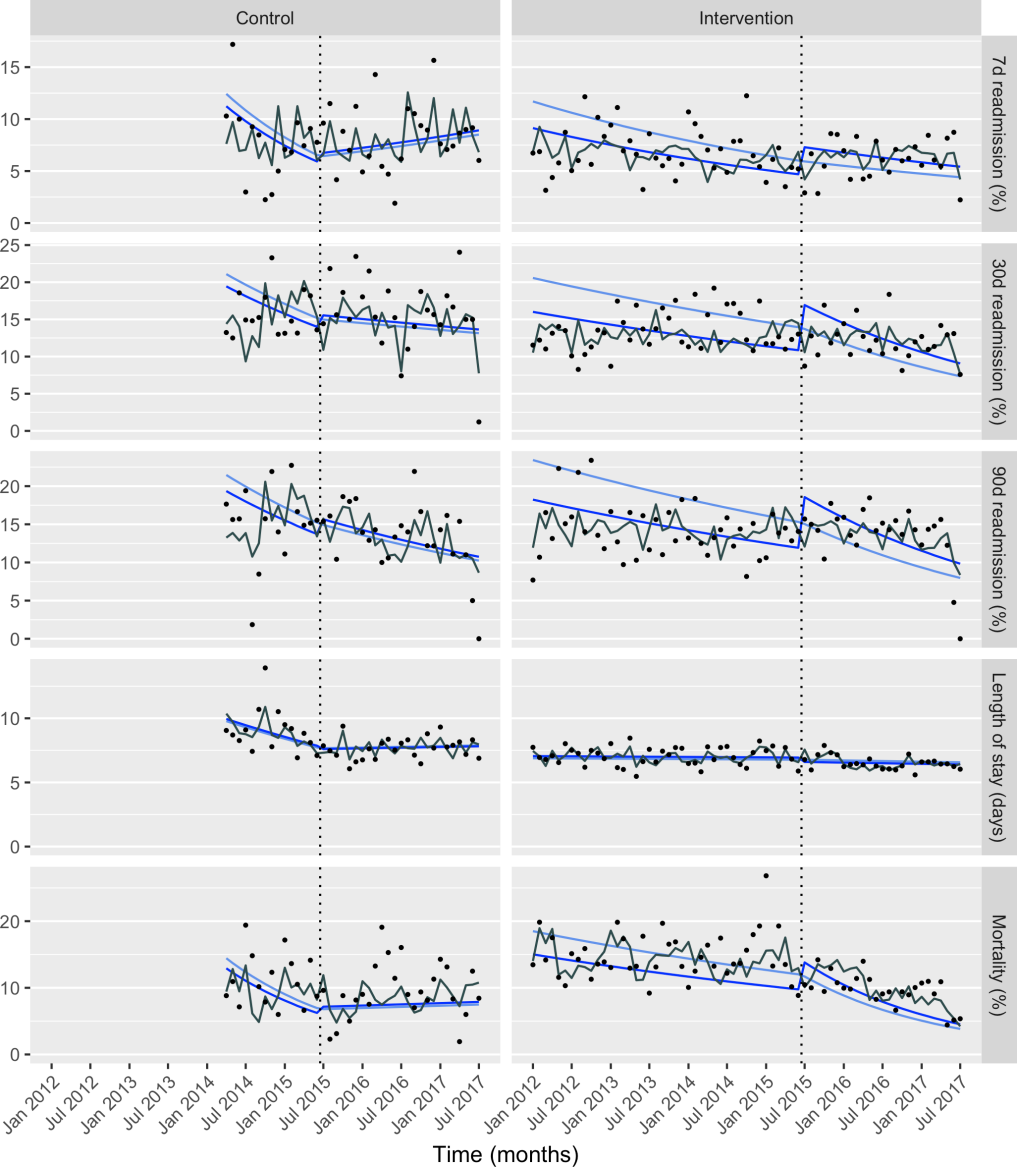
Interrupted time series analysis of heart failure admission outcomes. Time series with continuous time trends pre-intervention and post-intervention (heart failure care bundle) for control and intervention hospital sites. Circular markers for each month denote the aggregated measure for that month: in the case of readmissions and mortality, the proportion, in the case of length of stay, the geometric mean. The solid light blue line shows the fitted result of each model, on each site, with all covariates averaged over the entire study period; thus, this line is guaranteed to be continuous at the interruption by the use of linear splines. The solid dark blue line shows the fitted result for the pre-intervention and post-intervention periods, on each site, with all covariates averaged within each period (pre-intervention and post-intervention). This line is not necessarily continuous across the interruption, since these average values differ between the two periods. The solid grey line shows the fitted result with covariates averaged over each month. Fitted results shown by these three solid lines were marginalised over random effects.

In what follows, all results are derived from the multivariable regression models and are adjusted for covariates as described in the Methods section. In particular, all trend effects are adjusted for changes in these covariates over the duration of the study. There were statistically significant changes of trend on the control site for 7-day readmissions, length of stay and mortality. This posed a serious issue for the intervention-control analysis, since we did not have data to explain this change. Therefore, we present results of the ITS for the intervention site as the main analysis, with separate analysis of the control site for context (table 3 and figure 2). Results of the intervention-control analysis are provided in online supplemental appendix B, but are not used in drawing conclusions on the effectiveness of the intervention. A Q–Q plot for the log-linear length of stay regression model is shown in online supplemental figure 1.

10.1136/bmjqs-2022-015511.supp2 Supplementary data

10.1136/bmjqs-2022-015511.supp3 Supplementary data

10.1136/bmjqs-2022-015511.supp4 Supplementary data

**Table 3:** Results of interrupted time series analyses

Outcome		Pre-intervention slope	Slope change ratio	Post-intervention slope
All-cause readmissions within 7 days Poisson regression: slopes are monthly incidence rate ratios	Control site	0.96 (0.92 to 0.99) p=0.01*	1.06 (1.01 to 1.11) p=0.02*	1.01 (1.00 to 1.03) p=0.17
	Intervention site	0.98 (0.98 to 0.99) p<0.001***	1.00 (0.97 to 1.02) p=0.66	0.99 (0.98 to 1.00) p=0.04*
All-cause readmissions within 7–30 days Poisson regression: slopes are monthly incidence rate ratios	Control site	0.98 (0.95 to 1.00) p=0.08	1.02 (0.98 to 1.05) p=0.31	0.99 (0.98 to 1.01) p=0.38
	Intervention site	0.99 (0.99 to 1.00) p<0.001***	0.98 (0.97 to 1.00) p=0.01*	0.97 (0.97 to 0.98) p<0.001***
All-cause readmissions within 30–90 days Poisson regression: slopes are monthly incidence rate ratios	Control site	0.98 (0.95 to 1.00) p=0.07	1.01 (0.97 to 1.04) p=0.62	0.98 (0.97 to 1.00) p=0.02*
	Intervention site	0.99 (0.99 to 0.99) p<0.001***	0.98 (0.97 to 1.00) p=0.004**	0.97 (0.97 to 0.98) p<0.001***
Length of stay Log-linear regression: slopes are monthly ratios	Control site	0.98 (0.97 to 0.99) p<0.001***	1.02 (1.01 to 1.03) p=0.005**	1.00 (1.00 to 1.01) p=0.61
	Intervention site	1.00 (1.00 to 1.00) p=0.62	1.00 (1.00 to 1.00) p=0.64	1.00 (1.00 to 1.00) p=0.32
In-hospital mortality Poisson regression: slopes are monthly incidence rate ratios	Control site	0.95 (0.92 to 0.98) p<0.001***	1.06 (1.02 to 1.10) p=0.007**	1.00 (0.99 to 1.02) p=0.60
	Intervention site	0.99 (0.98 to 0.99) p<0.001***	0.96 (0.95 to 0.98) p<0.001***	0.95 (0.95 to 0.96) p<0.001***

Results of interrupted time series analyses for 7-day, 30-day and 90-day all-cause readmission rates, length of stay and in-hospital mortality for patients discharged following an admission with heart failure. All models enforce continuity in time trends across the interruption. For each model, the following estimates are shown with 95% CIs and p values (*0.01≤p<0.05, **0.001≤p<0.01, ***p<0.001): pre-intervention slope compared with no trend, difference in slope from pre-intervention to post-intervention, post-intervention slope compared with no trend. The resulting slopes for the control site post-intervention, the intervention site during pre-intervention and the intervention site post-intervention are also shown, along with the corresponding differences in slope. Since all models were multiplicative due to the log link, all differences are ratios.

#### Main analysis: ITS, intervention site

##### HF diagnoses

Preliminary analysis on all emergency admissions to the intervention site (n=152 920), to investigate whether there were any differences in coding pre-intervention and post-intervention, showed that there was a small but statistically significant increase in the rate of spells with HF diagnoses post-intervention (incidence rate ratio (IRR) for post-intervention trend 1.01 (95% CI 1.01 to 1.02)). There was also a step change at the time of intervention (IRR 0.91 (95% CI 0.84 to 0.99)). The pre-intervention trend was essentially flat.

##### Outcome measures

There was no difference in trend for 7-day readmissions pre-intervention and post-intervention (ratio of rate ratio (RRR) 1.00, 95% CI 0.97 to 1.02). Trends in readmissions 7–30 and 30–90 days showed a statistically significant reduction post-intervention (both with RRR 0.98, 95% CI 0.97 to 1.00). The trend in mortality showed a statistically significant reduction post-intervention (RRR 0.96, 95% CI 0.95 to 0.98). There was no change in trend of length of stay post-intervention (RRR 1.00, 95% CI 1.00 to 1.00).

##### Intervention-control analysis

The results of the intervention-control analysis are similar to those of the intervention site analysis (online supplemental appendix B). A major exception is the length of stay analysis, which shows a comparative reduction in trend in length of stay for intervention versus control, driven by a downward trend on the control site during the baseline period that did not persist into the intervention period (clearly visible in figure 2). This exemplifies the issue with this secondary analysis, since such difference cannot reasonably be attributed to the intervention on this evidence alone. In the intervention-control analysis, the 7-day readmission trend reduction for intervention compared with control was statistically significant, and the 90-day trend reduction was not, although the effects were in the same direction in both analyses. The results for mortality were similar in both analyses.

##### Sensitivity analyses

The sensitivity analysis adjusting for IMD was consistent with the findings of the main analysis. The sensitivity analysis relaxing the continuity constraint on the time trends showed minimal differences from the main analysis, except for the difference in trend of 7-day readmissions (RRR 1.03, 95% CI 1.00 to 1.07).

## Discussion

The aim of the LNWUH HF care bundle project was to improve health, quality of life and experience of care for patients with acute HF. Following implementation of the care bundle, improvements in rates of echocardiography and specialist input were seen, in line with national guidelines. This study focused on the impact of the care bundle intervention on rates of emergency readmission to hospital following discharge, length of stay in hospital and in-hospital mortality. On the intervention site, trends in all-cause emergency readmissions 7–30 and 30–90 days were 2% lower post-intervention compared with baseline, while the trend in 7-day readmissions was unchanged. The trend in mortality on the intervention site showed a greater reduction, by 4%, post-intervention compared with baseline. There was no change in trend for length of stay on the intervention site.

National audit data show that compliance with care processes targeted by the bundle improved on the intervention site over the implementation period, in particular rates of echocardiography and specialist team input. The explicit focus on long-term success taken by the implementation team may have contributed to the success of this initiative, and in August 2022, the care bundle is still in use at LNWUH. The work that the team undertook mapping the echocardiography service at the intervention site led to additional funding through a Commissioning for Quality Improvement and Innovation target, totalling £1.2 million, to support the ongoing change. The team continues to negotiate resourcing to support HF care, including recruiting four full-time clinical nurse specialists in 2018/2019.

The observed improvements in readmissions 7–30 days and 30–90 days and mortality are plausibly a result of better care delivered through more timely and appropriate diagnosis and treatment of patients on admission to hospital with acute HF, although these effects were likely mediated through other aspects of the care pathway, such as prescription of evidence-based medications, in particular triple therapy.^26^

This study showed that a care bundle approach drawing on evidence-based interventions was associated with improvements in care, reductions in mortality and reductions in readmissions between 7 and 90 days post-discharge, without any increase in length of stay in hospital. These findings are relevant both in terms of improving the quality of care for patients with HF internationally, and more broadly given the scale of known issues in implementing evidence-based interventions into practice. Reducing or avoiding readmissions is beneficial for individual patients, and reduces demand if done appropriately. Strategies which support a reduction in readmission rates, and particularly early readmission which may relate to suboptimal inpatient management, are therefore worthy of attention.

A systematic review of hospital-based quality improvement interventions found inconsistent evidence, of very low to moderate quality, on effect of quality improvement initiatives on readmissions and mortality for HF.^27^ A randomised clinical trial showed that an educational intervention did not improve time to first rehospitalisation or death.^28^ Some studies did report an improvement, although they did not use a control group and therefore secular trends cannot be excluded.^29–31^ A systematic review identified increased patient understanding, self-care, including with involvement of carers and health professionals, and increased psychological well-being as key mechanisms of HF disease management interventions. Such approaches might effectively complement the care bundle approach taken in this study, with potential for further improvements.

The NHS, and health systems internationally, were placed under significant strain by the COVID-19 pandemic, and while in some ways health systems have changed and improved since the time period of this study, some of the same problems remain challenging. The National HF Audit covering 2020/2021 reported that a number of quality metrics appear to have been compromised in this recent period, including fewer hospitals achieving the echocardiography target, a fall in timely specialist follow-up and in referral rates to rehabilitation.^32^ The report also identified that access to diagnostics, cardiology care and beds needs to be improved for women and older patients. The approach used in this study could be of considerable benefit to providers seeking to make improvements in these areas.

More generally, this study demonstrates the feasibility of using a care bundle approach in improving hospital-wide outcomes for patients with chronic disease. It also provides an example of a robust observational design for evaluating the effectiveness of a care bundle. Many such initiatives are not robustly evaluated.^33^ Other quality improvement initiatives could adopt this approach, combining a quality improvement initiative with an ITS design, to ensure learning is captured and shared.

### Strengths and limitations

The quasi-experimental study design, ITS with controls, is among the strongest of observational study designs.^18^ This design is particularly appropriate for care bundle interventions in which the evidence base for the elements is already established. In this case, the care bundle elements were drawn from national (NICE) guidelines, and the study aim is to evaluate the effectiveness of an attempt to improve compliance. The use of a control site serving a population with similar demographics is a strength of this design; however, it is important to note that the two sites were not perfectly comparable, and control site admission data were not available for all of the pre-intervention period. Furthermore, over the study period, we cannot rule out changes to care delivery at the control site, and indeed the outcome measures of interest did change at the control site over the duration of the study. As a result, we used intervention site-only ITS analysis as the main analysis in this study, to avoid conflating the impact of the intervention with independent changes at the control site. This meant that in this study, the full benefits of the ITS with controls design were not realised. The analysis was adjusted for age, gender, ethnicity and, in a sensitivity analysis, socioeconomic deprivation. However, from the available data, we were unable to adjust for other variables such as case severity and comorbidity. While it is unlikely these variables were systematically different over the duration of the study, we cannot rule this out completely. This design, in combination with the use of process, outcome and balancing measures, as well as the systematic approach to quality improvement, conveys confidence in the findings of this study, as well as supporting learning during the initiative.

## Conclusion

The improvement team at NPH achieved improvements in the quality of patient care associated with implementing an HF admission care bundle to change systems and processes of care. For patients admitted with acute HF, these improvements in care were associated with reductions in in-hospital mortality and readmission rates 7–90 days post-discharge, without associated increases in length of stay in hospital. Other hospitals seeking to improve care and outcomes for patients with HF should consider adopting a care bundle approach to implementing key elements of national care guidelines. Quality improvement initiatives should consider adopting a systematic approach to improvement, and a robust observational study design using multiple control sites, with comprehensive data on care processes and context across sites. Future research should focus on better understanding the mechanisms through which improvements in care on admission impact on downstream outcomes, and on the longer-term implications of these improvements, especially in relation to care in the community post-discharge.

## Data Availability

No data are available.
