# Supplementary material for: Effect of implementing a heart failure admission care bundle on hospital readmission and mortality rates: interrupted time series study
Source: BMJ Qual Saf. 2023 Nov 5;33(1):55–65. doi: 10.1136/bmjqs-2022-015511 (PMC10804004; doi:10.1136/bmjqs-2022-015511)
Supplement: Supplementary data [file bmjqs-2022-015511supp001.pdf]

Appendix A

Supplementary table 1: Quality Improvement Methods used in implementation of the heart failure care bundle

**Method**<br>**Strategic principle(s): and simple rule(s) of the SHIFT-Evidence framework* [1]	**Description and Purpose**	**Findings**
Measurement for Improvement [2]<br><br>*Act Scientifically and Pragmatically: Assess whether improvement is achieved, and capture and share learning*	A few simple and specific measures, linked to your objectives and aims, and available in real time to demonstrate whether changes are making improvements.	Regular reporting on bundle implementation helped the team monitor their progress and keep staff on the wards engaged. Displaying weekly data on wards and highlighting results to staff helped drive improvement, especially for referral to the HF specialist.
Long term Success Tool[3]<br><br>*This method is relevant to nearly all simple rules in the SHIFT-Evidence framework*	The tool allows teams to reflect upon factors known to influence long term success of improvement initiatives. Team members enter scores quarterly on WISH. They individually score 12 factors that have been identified to impact long term success. They also have the opportunity within the tool to highlight any potential actions or comments for specific factors.	LTS- helped focus efforts around stakeholders and what people are thinking about the project. Most recent results showed the highest risk is perceived to be around resources to keep the project going.
Patient and Public Engagement and Involvement[4]<br><br>*Engage and Empower: Actively engage those responsible for and affected by change, Facilitate dialogue*	4PI<br>Promote clarity on the purpose of involvement, the individuals and groups who will be present and their role in the process.	Patients involved in the team helped champion ideas to improve patient experience. The team focused on getting more information to patients. British Heart Foundation material along with hospital specific material were developed and tested with patients to get their perspective on the information they receive while in hospital and how this could be improved.
Action Effect Diagram[5]<br><br>*Act Scientifically and Pragmatically: Understand problems and opportunities*	The articulation of an overall aim, potential interventions that will be tested in an attempt to achieve this aim, hypothesised cause/effect	The team iterated their initial AED to reflect their work around ECHO, specialist input, referrals and medication reconciliation.

	relationships linking interventions to the aim along with measures to support evaluation	
Stakeholder Mapping<br><br>*Engage and Empower: Actively engage those responsible for and affected by change, Facilitate dialogue, Foster a culture of willingness to learn and freedom to act*	Actively engaging a wide variety of people such as clinicians, administrative staff, patients and user groups will help you deliver your change project. Stakeholder mapping enables you to identify everyone who needs to be involved and assess how much time and resource to give to maintaining their involvement and commitment.	The project has highlighted key working relationships and how to work well across boundaries, including working beyond clinical hierarchies.
Process Mapping[6]<br><br>*Embrace Complexity: Understand processes and practices of care*	Process mapping provides insight into systems and processes in which improvement interventions are introduced and is seen as useful in healthcare quality improvement projects.	The team mapped the process by which patients were identified as needing the care bundle, and how patients were admitted and reviewed within the clinical pathway.<br><br>The process mapping exercise highlighted the need for ECHO service redesign. The team developed a business case and secured more equipment and staff for the ward for this service to continue. This increased the service's capacity for ECHO, including at weekends.
Plan, Do, Study, Act (PDSA) cycle[7]<br><br>Act Scientifically and Pragmatically: Identify, test and iteratively develop potential solutions	Translation of ideas and intentions into action. Provides a structured experimental learning approach to testing changes.	Project team completed over 40 PDSA cycles during the implementation period, documenting these cycles on the WISH platform. Three new wards opened in January, necessitating changes to the system the team had in place over the first 6 months of testing the bundle. The team used PDSAs to test approaches to resolving this, including additional awareness raising and training for new staff on these wards.

** the mapping suggested here should not be taken as exhaustive or representative: each method can be associated with multiple principles and simple rules, only the most directly relevant are listed here*

References

1. Reed JE, Howe C, Doyle C, Bell D. Simple rules for evidence translation in complex systems: A qualitative study. *BMC Med*. 2018;16(1):92. doi:10.1186/s12916-018-1076-9

2. Provost LP. Analytical studies: a framework for quality improvement design and analysis. *BMJ Qual Saf*. 2011;20(Suppl 1):i92-i96. doi:10.1136/bmjqs.2011.051557

3. Lennox L, Doyle C, Reed JE, Bell D. What makes a sustainability tool valuable, practical and useful in real-world healthcare practice? A mixed-methods study on the development of the Long Term Success Tool in Northwest London. *BMJ Open*. 2017;7(9):e014417. doi:10.1136/bmjopen-2016-014417

4. Faulkner A, Yiannoullou S, Kalathil J, et al. *Involvement for Influence: 4PI National Involvement Standards*.; 2015.

5. Reed JE, McNicholas C, Woodcock T, Issen L, Bell D. Designing quality improvement initiatives: the action effect method, a structured approach to identifying and articulating programme theory. *BMJ Qual Saf*. 2014;23(12):1040-1048. doi:10.1136/bmjqs-2014-003103

6. Antonacci G, Reed JE, Lennox L, Barlow J. The use of process mapping in healthcare quality improvement projects. *Heal Serv Manag Res*. 2018;31(2):74-84. doi:10.1177/0951484818770411

7. Taylor MJ, McNicholas C, Nicolay C, Darzi A, Bell D, Reed JE. Systematic review of the application of the plan-do-study-act method to improve quality in healthcare. *BMJ Qual Saf*. 2014;23(4):290-298. doi:10.1136/bmjqs-2013-001862
