# Supplementary material for: Effect of implementing a heart failure admission care bundle on hospital readmission and mortality rates: interrupted time series study
Source: BMJ Qual Saf. 2023 Nov 5;33(1):55–65. doi: 10.1136/bmjqs-2022-015511 (PMC10804004; doi:10.1136/bmjqs-2022-015511)
Supplement: Supplementary data [file bmjqs-2022-015511supp002.pdf]

Appendix B

Supplementary table 2: *Results of interrupted time series analyses with discontinuous time trends*

Caption: Results of interrupted time series analyses for 7-, 30- and 90-day all-cause readmission rates, length of stay and in-hospital mortality for patients discharged following an admission with heart failure. For each intervention-control model, the following estimates are shown with 95% confidence intervals and p-values: pre-intervention slope on the control site compared with no trend, difference in slope from pre- to post-intervention on the control site, difference in slope from control site to intervention site during the pre-intervention period and the difference in difference in slope between control and intervention sites, pre- and post-intervention. The resulting slopes for the control site post-intervention, the intervention site during pre-intervention, and the intervention site post-intervention, are also shown, along with the corresponding differences in slope.

In addition, results are shown for intervention-site-only models for each outcome. This acts as a sensitivity analysis for the relaxation of the time trend continuity constraint in the main analysis. For these intervention-site-only models, the following regression coefficients are given with 95% confidence intervals and p-values: pre-intervention slope (on the intervention site), the difference in slope from pre- to post-intervention (on the intervention site). The resulting post-intervention slope (on the intervention site) is also given. Since all models were multiplicative due to the log link, all differences are ratios.

Outcome		Pre-intervention slope	Slope change ratio	Post-intervention slope
All-cause readmissions within 7 days<br><br>Poisson regression: slopes are monthly incidence rate ratios	Control site	0.93 (0.89 – 0.98)<br>p = 0.004	1.08 (1.02 – 1.13)<br>p = 0.005	1.00
	Slope change ratio	1.06 (1.01 – 1.11)<br>p = 0.02	0.93 (0.88 – 0.99)<br>p = 0.014	0.99
	Intervention site	0.99	1.01	0.99
	Intervention site (no control)	0.95 (0.92 – 0.99)<br>p = 0.005	1.03 (1.00 – 1.07)<br>p = 0.08	0.98
All-cause readmissions within 7-30 days<br><br>Poisson regression: slopes are monthly incidence rate ratios	Control site	0.96 (0.93 – 0.99)<br>p = 0.009	1.04 (1.00 – 1.08)<br>p = 0.052	0.99
	Slope change ratio	1.04 (1.00 – 1.07)<br>p = 0.033	0.95 (0.92 – 0.99)<br>p = 0.014	0.99
	Intervention site	0.99	0.99	0.98
	Intervention site (no control)	0.99 (0.99 – 1.00)<br>p = 0.013	0.99 (0.98 – 1.00)<br>p = 0.046	0.98

All-cause readmissions within 30-90 days<br><br>Poisson regression: slopes are monthly incidence rate ratios	Control site	0.96 (0.93 – 0.99)<br>p = 0.016	1.03 (1.00 – 1.07)<br>p = 0.073	0.99
	Slope change ratio	1.02 (0.99 – 1.06)<br>p = 0.13	0.97 (0.93 – 1.00)<br>p = 0.06	0.99
	Intervention site	0.99	1.00	0.98
	Intervention site (no control)	0.99 (0.98 – 0.99)<br>p < 0.001	0.98 (0.97 – 0.99)<br>p < 0.001	0.97
Length of stay<br><br>Log-linear regression: slopes are monthly ratios	Control site	0.99 (0.98 – 1.00)<br>p = 0.033	1.01 (1.00 – 1.03)<br>p = 0.028	1.00
	Slope change ratio	1.01 (1.00 – 1.02)<br>p = 0.042	0.99 (0.97 – 1.00)<br>p = 0.027	1.00
	Intervention site	1.00	1.00	1.00
	Intervention site (no control)	1.00 (1.00 – 1.00)<br>p = 0.6	1.00 (1.00 – 1.00)<br>p = 0.62	1.00
In-hospital mortality<br><br>Poisson regression: slopes are monthly incidence rate ratios	Control site	0.94 (0.91 – 0.98)<br>p = 0.003	1.07 (1.02 – 1.11)<br>p = 0.003	1.01
	Slope change ratio	1.05 (1.01 – 1.10)<br>p = 0.01	0.92 (0.88 – 0.96)<br>p < 0.001	0.96
	Intervention site	0.99	0.98	0.97
	Intervention site (no control)	0.99 (0.99 – 1.00)<br>p = 0.008	0.97 (0.96 – 0.99)<br>p < 0.001	0.97
