# Supplementary material for: Effect of implementing a heart failure admission care bundle on hospital readmission and mortality rates: interrupted time series study
Source: BMJ Qual Saf. 2023 Nov 5;33(1):55–65. doi: 10.1136/bmjqs-2022-015511 (PMC10804004; doi:10.1136/bmjqs-2022-015511)
Supplement: Supplementary data [file bmjqs-2022-015511supp003.pdf]

Supplementary Figure 1 Caption: Normal Q-Q plot for regression model for logarithm of length of stay.
